# Zirconium Dental Implants as Potential Optical Waveguides in Photodynamic Inactivation of Bacterial Biofilms—A Pilot Study

**DOI:** 10.3390/microorganisms13040850

**Published:** 2025-04-08

**Authors:** Kolja Lehmann, Gabor Kadler, Alexander Kalyanov, Tiziano A. Schweizer, Heinrich Walt, Harald Essig

**Affiliations:** 1 Department of Cranio-Maxillo-Facial and Oral Surgery, University Hospital Zurich, University of Zurich, 8091 Zurich, Switzerland; koljakarim.lehmann@uzh.ch (K.L.); gabor.kadler@usz.ch (G.K.);; 2 Biomedical Optics Research Laboratory, University Hospital Zurich, University of Zurich, 8091 Zurich, Switzerland; 3 Department of Dermatology, University Hospital Zurich, University of Zurich, 8091 Zurich, Switzerland

**Keywords:** periimplantitis, zirconium dental implants, titanium dental implants, photodynamic inactivation (PDI), One Health approach, *Staphylococcus epidermidis*, biofilm

## Abstract

In patients with predisposing risk factors, bacterial colonization of dental implants can lead to periimplantitis (PI). Established individual treatment protocols can be effective, but antimicrobial resistance (AMR) and biofilm formation may impede successful treatment, therefore requiring surgical intervention. Photodynamic Inactivation (PDI) combined with optical waveguides could eradicate such pathogens without the risk of new AMR emergence and reduce the need for surgery. In this pilot study, we investigated the waveguiding function of light-transmitting zirconium dioxide (ZrO_2_) dental implants of different diameters by quantifying their transmission spectrum, fraction of transmitted red-light intensity, and potential polarizing properties. In addition, PDI experiments involving in vitro grown *Staphylococcus epidermidis* biofilms on ZrO_2_ and titanium alloy (TAV) discs were performed. Colonized discs were treated with Methylene Blue (MB) photosensitizer before red-light illumination (670 nm) at various intensities. A reduction in bacterial colony-forming units (CFUs) of up to 85% was observed on ZrO_2_ discs. Meanwhile, the biofilms grown on TAV discs showed no significant reduction in CFUs. These findings make ZrO_2_ a potential candidate in augmentative PDI treatment of PI. The successful use of PDI combined with waveguiding ZrO_2_ dental implants can support the reduction in antibiotic prescriptions, thus advancing the WHO’s One Health approach of antibiotic stewardship.

## 1. Introduction

Progress in surgical procedures and development of dental implant material has led to a constant increase in the number of inserted implants in dentistry. However, certain complications still need to be addressed to ensure optimal oral rehabilitation. An important complication is periimplantitis (PI), which describes a bacterial infection of dental implants and adjacent tissue [1,2]. Association with *Staphylococcus epidermidis* and typical species in periodontitis, such as *Porphyromonas gingivalis* or *Tannerella forsythia*, was shown for PI [3,4]. The foreign substance of the artificial dental implant poses a suitable environment for bacteria to accumulate and form biofilm structures, eventually leading to PI [1,5,6]. While the implant surface roughness correlates with the desired bone fixation, it also favors bacterial retention, and the implant itself might trigger inflammatory immune reactions [7,8,9]. Conventionally used dental implant materials like titanium alloys (TAV) or zirconium dioxide ceramics (ZrO_2_) are both affected by PI, without any known differences in biofilm accumulation between them [7].

PI occurrence is linked to plaque biofilm formation, and risk factors such as smoking, diabetes mellitus, or poor oral hygiene are being discussed [6,10]. Treatment should consider the regional extent of the infection and the involved pathogen’s antimicrobial resistance (AMR) [6,11]. Depending on the individual PI progression, therapy strategies might range from conservative application of chlorhexidine and prescription of antibiotics to extensive surgical debridement of necrotic mucosa and bone tissue [12,13,14]. In severe cases, subsequent reconstructive surgery may be needed, with the risk of surgical complications and prolonged healing durations [12,13,15]. Bacterial biofilm dispersed around the whole implant surface, including concealed spots at the intraosseous tip, may hinder complete remission from inflammation of the surrounding soft tissue and bone matrix [16,17]. Without preventative measures and early intervention, a removal of the implant is often imminent [18]. Furthermore, the biofilm matrix confers protection from pharmaceuticals, thus resulting in an up to thousand-fold higher antibiotic tolerance that may promote AMR formation [19,20]. Simultaneously, the global prevalence of beta-lactam-resistant *Staphylococcus* species is unprecedentedly rising, posing a substantial risk for future infection therapy outcomes [21,22]. The WHO’s One Health approach to reduce the utilization of antibiotics may be key to combating the associated future disease burden and optimizing the outcome in PI [22].

To target the complex PI development, this pilot study focused on a new non-invasive Photodynamic Inactivation (PDI) approach. PDI could augment current PI treatment protocols to optimize the outcome and even prevent its onset. It is already in clinical use to treat dermatological basal cell carcinoma and dental periodontitis [23,24,25]. Studies could show that it does not cause AMR formation and is therefore in line with the WHO’s One Health approach [26]. It relies on non-ionizing visible or near-infrared light to activate a dissolved photosensitizer (PS) molecule. By absorbing the light, PS electrons are pushed to an excitation state [23]. From there, the electrons (Type 1 reaction) or the excitation energy (Type 2 reaction) is arbitrarily transferred to surrounding oxygen molecules, forming cytotoxic oxygen radicals (ROS) [27]. A frequently used PS in PDI is the cationic phenothiazinium dye Methylene Blue (MB) [24,28]. It can perform both Type 1 and Type 2 reactions and has a maximum absorption in the visible red-light spectrum at 664 nm [29]. The reaction sequence of PDI with MB proved to be effective in destructing bacterial biofilms on exogen implant materials, leading to the potential application in treating PI [15,30].

Despite its bactericidal effect, the challenge of reaching the concealed intraosseous biofilm formation around the dental implant remains unsolved. Implementing a dental implant with waveguiding properties could present a significant step towards transmitting the required light intensity to the intraosseous biofilms. ZrO_2_ dental implants are known to show such light-transmission features due to their ceramic material properties [31,32]. Previous studies showed a similar accumulation of biofilm on ZrO_2_ and TAV, as well as the promising bactericidal effect of PDI [33,34]. This study therefore envisioned assessing the potential of ZrO_2_ dental implants as waveguides for PDI and their consequential advantage over TAV in an in vitro setting.

This pilot study conducted novel fundamental research regarding PDI with ZrO_2_ as optical waveguides to determine their potential clinical application in PI treatment. The waveguiding capabilities of ZrO_2_ are envisioned to support the antibacterial effect of PDI. To test this hypothesis, ZrO_2_ discs were cultivated with *S. epidermidis* biofilms, and in vitro PDI with MB was performed to determine the bactericidal effect. Testing a range of MB concentrations and light intensities allowed for the optimal treatment combination to be determined. The sole effect of the light-transmission property in ZrO_2_ discs was quantified by reproducing the same experiments with non-transparent titanium (TAV) discs. Simultaneously, the light properties of ZrO_2_ dental implants were tested in separate physical light experiments to quantify their waveguiding function for further in-depth research.

## 2. Materials and Methods

### 2.1. Optical Experiments on Zirconium Dental Implants

**Figure 1:**
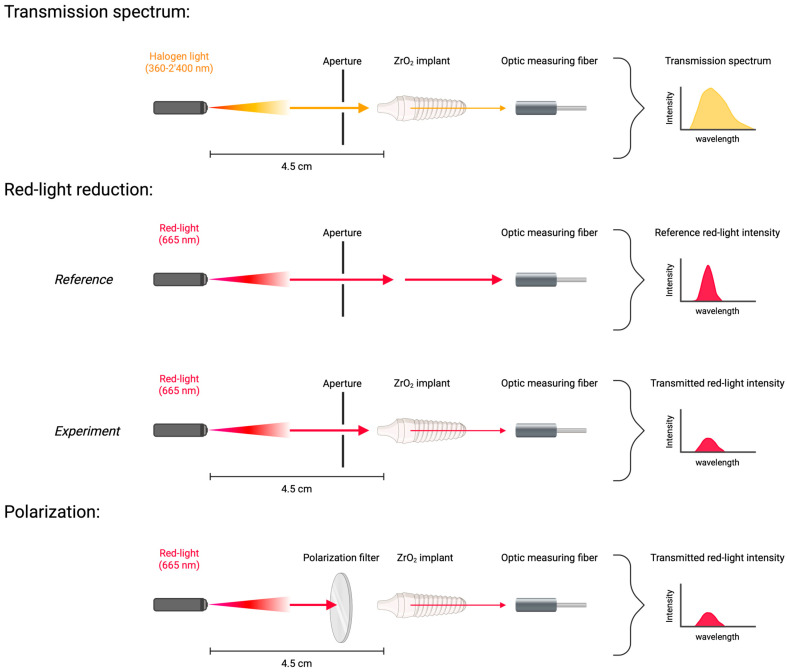
Visualization of the conducted optical experiments. ZrO_2_—zirconium dioxide ceramics. Yellow arrow—halogen light; red arrow—red-light. Created by Lehmann et al. (2025), Biorender.com (https://Biorender.com/tbdjb45, accessed on 4 April 2025).

In preliminary optical investigations, ZrO_2_ showed visible-light transmission, while TAV was confirmed to be non-transparent (Supplementary Figures S1–S3). Therefore, the conducted optical experiments focused on ZrO_2_ dental implants. The waveguiding function of ZrO_2_ dental implants (Zircon Medical AG, Altendorf, Switzerland) was investigated by determining the transmission spectrum and measuring the red-light intensity reduction through such implants (Figure 1). All experiments were performed in absolute darkness to eliminate the distorting background light, and apertures were implemented to reduce scattered radiation. For the transmission spectrum, a ZrO_2_ dental implant (length: 11 mm, diameter: 4.5 mm) was illuminated along its longitudinal axis with a halogen light source of 360–2400 nm wavelength (HL-2000, Ocean Optics, Orlando, FL, USA) at a distance of 4.5 cm. The transmitted light was detected with an optical measuring fiber (M53L02 SMA Fiber Patch, Thorlabs, Newton, NJ, USA) that was positioned directly behind the implant. The red-light intensity reduction was measured with ZrO_2_ dental implants that were identical in shape, texture, and length (11 mm) but differed in diameter (3.5, 4.1, 4.5, and 5 mm). A 670 nm red-light source (Red Mini 670, Red Light Man Ltd., Manchester, UK), in combination with neutral density filters (optical density ranging from 1 to 4, Thorlabs, Newton, NJ, USA), was implemented to regulate the light intensity of incoming longitudinal illumination onto the different implants. The distance between the light source and implant was 4.5 cm, and the same optic fiber measured the fraction of transmitted red-light intensity. To quantify the red-light reduction, the intensity of the red light itself was set as a reference, and the measured intensities were normalized relative to it. Further, the red-light source was used to investigate the light polarization through ZrO_2_ dental implants. Polarization filters (diameter, 49 mm; HOYA Corporation, Tokyo, Japan) were positioned between the light source and a ZrO_2_ implant (length, 11 mm; diameter, 4.5 mm) (Figure 1). During the experiment, the polarization filters were manually rotated into predefined positions (0°, 45°, 90°, 135°, and 180° degrees) to change the oscillation direction of incoming red light. After the positioning of the filter, the implant was illuminated with red light, and the previously used optic fiber measured the transmitted red-light intensity behind the dental implant. The obtained intensity values from each position angle were compared to detect any deviation that would indicate a light polarization through ZrO_2_ dental implants.

### 2.2. Photodynamic Inactivation (PDI) of Bacterial Biofilm

The study protocol involved a previously isolated *Staphylococcus epidermidis* strain (BCI195) that was approved by the Cantonal Ethics Commission (KEK) in Zurich, Switzerland (KEK No. 2016-00145 and No. 2017-01458). As PI involves persistent bacterial biofilm processes, the obtained *S. epidermidis* strain was used to grow biofilms around sterilized implant discs [16]. The biofilm was subsequently destructed with PDI, relying on an MB solution (Methylene Blue solution, 50484-100ML-F, Sigma Aldrich, St. Louis, MO, USA) as a photosensitizer. The two used discs’ materials were the waveguiding zirconium dioxide ceramic (ZrO_2_) (Zircon Medical AG, Altendorf, Switzerland) and the non-transparent titanium alloy Ti-6AI-4V (TAV) (DePuy Synthes, Zuchwil, Switzerland). The non-transparent properties of TAV discs were visually verified in preliminary optical investigations (Supplementary Figures S1 and S2). All discs were uniform in regard to their round shape (diameter, 10 mm).

#### 2.2.1. Biofilm Cultivation Process

**Figure 2:**
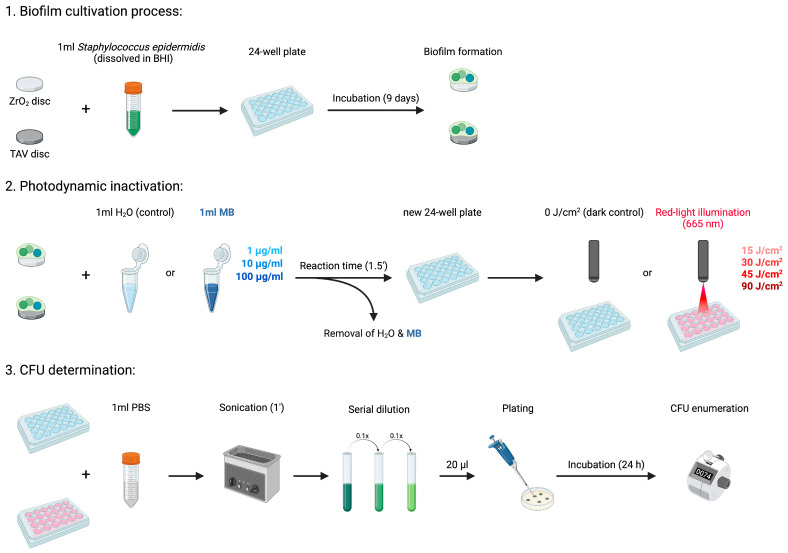
Visualization of the conducted PDI experiment sequence. ZrO_2_—zirconium dioxide ceramics; TAV—titanium alloy Ti-6AI-4V. Created by Lehmann et al. (2025), Biorender.com (https://BioRender.com/rp9q8g1, accessed on 4 April 2025).

A fresh *S. epidermidis* suspension was obtained by dissolving pre-grown bacteria colonies from the isolated strain in 5 mL Brain Heart Infusion (BHI) (Becton, Dickinson and Company, Heidelberg, Germany). This suspension was incubated for 24 h under aerobic conditions at 37 °C in a shaking incubator, with 100 rotations per minute (rpm). On the following day, the suspension was diluted by 1:2000 with BHI. Then, 2 mL of the diluted bacteria suspension was transferred onto ZrO_2_ or TAV discs in sterile 24-well plates to start the biofilm cultivation process (Figure 2). The discs were incubated for 6 days under aerobic conditions at 37 °C. BHI was exchanged after 3 days to guarantee optimal biofilm cultivation. Next, the liquid phase was removed. To eradicate residual planktonic bacteria, the biofilm cultivated implant discs were then transferred into new 24-well plates and rinsed twice with 1 mL of Phosphate-Buffered Saline (PBS) (Thermo Fisher Scientific, Waltham, MA, USA).

#### 2.2.2. In Vitro Photodynamic Inactivation Process

After the biofilm cultivation, the ZrO_2_ and TAV discs in the individual 24-well plates were assigned to series 1 and 2, forming technical duplicates. The two series represented technical replicates, increasing the validity of the experiment for later data analysis. A series consisted of multiple discs of the same material (ZrO_2_ or TAV). One disc within a series was always treated with 1 mL of pure water (H_2_O) as a negative control. The remaining discs were treated with 1 mL of 1, 10, or 100 µg/mL MB (Figure 2). After 90 s, water and MB solutions were removed, and the 24-well plates were assigned to a dark control or an intervention group. The dark control group was placed into complete darkness (0 J/cm^2^), while the intervention group underwent photodynamic illumination with the 670 nm red-light source previously implemented in the optical experiments. Illumination was conducted with one of the following four intensities: 15, 30, 45, and 90 J/cm^2^. The 30 J/cm^2^ intensity was achieved by illuminating with 15 J/cm^2^ from above and below, while the other intensities were accomplished by illuminating only from above. After PDI, the remaining bacteria were detached by irrigating all discs with 1 mL PBS and sonicating them for 1 min at 40 Hz. Next, 1 mL of the resulting solution from each disc was transferred to a sterile microplate. After dilution in tenfold steps, 20 µL of each dilution step was plated onto agar plates and incubated for 24 h under aerobic conditions at 37 °C. On the following day, the number of colony-forming units per mL (CFUs/mL) on each agar plate was visually enumerated (Figure 2). For each 24-well plate, the CFU from the H_2_O control duplicates represented the reference for bacterial growth without PDI. The values under PDI treatment were standardized on the corresponding reference. To determine the survival rate of *S. epidermidis* under PDI (denoted as CFU_rel_), the enumerated CFU value for each disc was divided by the CFU of the H_2_O control disc from the corresponding series within the 24-well plate.

### 2.3. Statistical Analysis

For each MB concentration (1, 10, and 100 µg/mL), the standardized CFU values for ZrO_2_ and TAV treated with different red-light intensities (15, 30, 45, and 90 J/cm^2^) were compared to the corresponding control group (0 J/cm^2^). Statistical analysis was performed using GraphPad Prism 10 (GraphPad Software Inc., Boston, MA, USA), R version 4.2.2 (R Foundation for Statistical Computing, Vienna, Austria), and conducted statistical tests are indicated in the figure descriptions. The statistical analysis relied on Welch’s *t*-test and the determination of the corresponding *p*-value for the involved technical duplicates from series 1 and 2.

## 3. Results

### 3.1. Zirconium Dental Implants Waveguiding Properties

**Figure 3:**
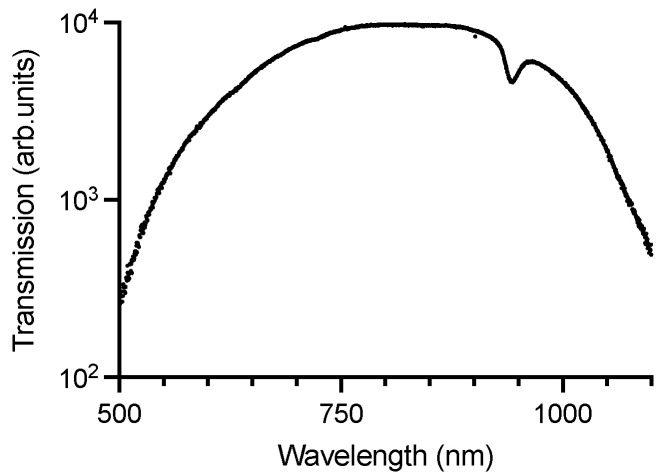
Transmission spectrum of ZrO_2_ dental implants illuminated with a halogen lamp. ZrO_2_—zirconium dioxide ceramics.

**Figure 4:**
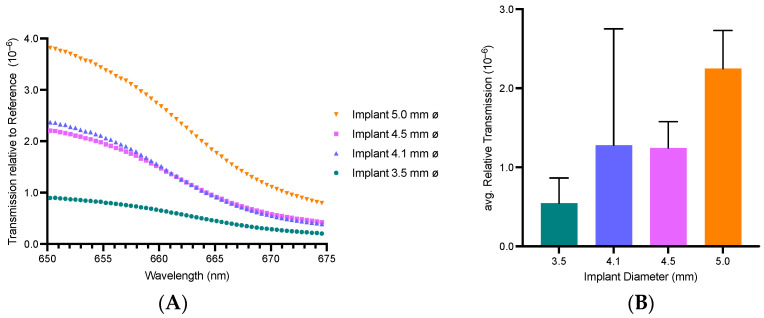
Transmitted light intensity for four ZrO_2_ implant diameters relative to the wavelength-dependent reference value (**A**). Corresponding average transmitted light intensity between 650 and 675 nm for each implant diameter (**B**). Underlying data can be found in Supplementary Table S1. Illuminated with Red Mini 670. ZrO_2_—zirconium dioxide ceramics.

ZrO_2_ dental implants are known to partially transmit visible and near-infrared light [31,32]. To quantify the transmission spectrum, a white halogen light source (wavelength range: 500–1100 nm) was used to illuminate the implants. The maximum transmission of ZrO_2_ dental implants can be observed in the infrared region at 822.19 nm (Figure 3). The wavelength 665 nm, used for the activation of MB Photosensitizer in PDI, shows a near-maximum transmission through ZrO_2_ implants, fostering the feasibility of their waveguiding function in PDI with MB. Therefore, subsequent optical experiments used a 670 nm light source to specifically quantify the red-light reduction through ZrO_2_ implants. Increased diameter shows increased light transmission converging towards 675 nm (Figure 4A). A disproportional large difference in transmitted intensity is evident when increasing the diameter from 3.5 to 4.1 and from 4.1 to 5 mm, respectively (Figure 4B). A positive correlation between implant diameter and transmitted light intensity can be observed. Light-intensity transmission disproportionally increased relative to the implants’ cross-sectional area (Supplementary Figure S4).

**Figure 5:**
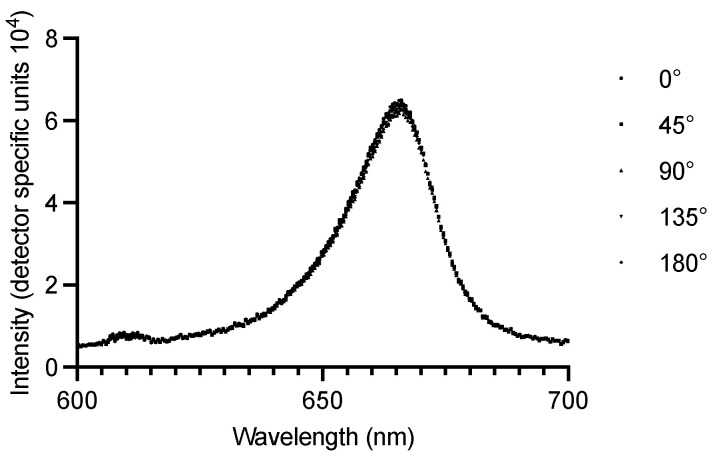
Light polarization through ZrO_2_ dental implants for different polarization filter-angle positions. ZrO_2_—zirconium dioxide ceramics.

Further, the light polarization through ZrO_2_ dental implants was investigated. ZrO_2_ dental implants transmit electromagnetic waves independent of their three-dimensional oscillation direction; thus, the transmitted light intensity stays unaffected (Figure 5). No polarization was found, and the transmitted red-light intensity remained constant. Additionally, it was visually observed that the ZrO_2_ implants homogenously scattered the 670 nm red light in all directions (Supplementary Figure S3).

### 3.2. PDI Biofilm Destruction Comparison ZrO_2_ and TAV

**Figure 6:**
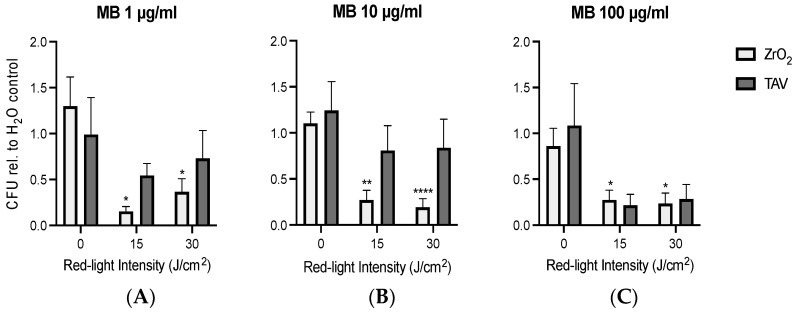
PDI with MB concentrations of 1 μg/mL (**A**), 10 μg/mL (**B**), and 100 μg/mL (**C**) of *Staphylococcus epidermidis* biofilm formations on ZrO_2_ and TAV discs, showing mean CFU values standardized to the corresponding CFU value from the H_2_O control group (CFU_rel_). The dark control group within an MB concentration for ZrO_2_ and TAV is depicted by 0 J/cm^2^. Statistical analysis was performed with Welch’s two-sample *t*-test, with asterisks indicating statistically significant *p*-values for CFU reduction relative to corresponding 0 J/cm^2^ value (* *p* < 0.05, ** *p* < 0.01, and **** *p* < 0.0001). Underlying data can be found in Supplementary Table S3. MB—Methylene Blue; ZrO_2_—zirconium dioxide ceramics; TAV—titanium alloy Ti-6AI-4V.

PDI has recently shown promising bactericidal effects, leading to a possible treatment option in PI [30]. This pilot study used PDI with the MB photosensitizer to reduce established *S. epidermidis* biofilm formations on ZrO_2_ and TAV discs (Figure 6). The reduction was measured via the calculated CFU_rel_ number to compare the PDI efficacy on the two dental implant materials. The 0 J/cm^2^ dark control group showed the possible intrinsic effect of MB on the bacterial growth in complete darkness. This intrinsic bactericidal effect was limited because the corresponding CFU_rel_ was confined to 1, with varying standard deviations. For one of the three MB concentrations (1, 10, and 100 µg/mL), increasing the light intensity generally led to lower CFU_rel_ numbers. Increasing the light intensity from 0 to 15 J/cm^2^ generally showed a stronger reduction in CFU_rel_ than doubling the intensity from 15 to 30 J/cm^2^. This holds true for all the MB concentrations tested here. Initially, higher light intensities of 45 and 90 J/cm^2^ were also tested with 10 µg/mL MB (Supplementary Figure S5 and Table S2).

PDI on ZrO_2_ led to visibly lower CFU_rel_ (ranging from 0.15 to 0.37) compared to the remaining larger CFU_rel_ values on TAV (ranging from 0.23 to 0.84). While the CFU_rel_ difference between 0 and 15 or 30 J/cm^2^ was statistically significant in all three MB concentrations for ZrO_2_, the CFU_rel_ difference for TAV was not significant in any (Supplementary Table S3). ZrO_2_ demonstrated a stronger CFU_rel_ reduction in MB 1 and 10 µg/mL compared to TAV. Interestingly, the strongest bactericidal effect was observed on ZrO_2_ in the most preserving PDI treatment protocol, with a low MB concentration of 1 µg/mL and a limited light intensity of 15 J/cm^2^. The resulting mean CFU_rel_ was 0.15, indicating an 85% reduction in CFUs compared to the corresponding 0 J/cm^2^ group.

## 4. Discussion

Effective PDI might improve PI treatment’s outcome by reducing bacterial biofilm survival [30]. Limited prognosis and globally increasing AMR formation demand innovative treatment options in line with the global One Health approach [21]. PDI with non-ionizing red light presents an augmentative treatment option for PI [35,36]. While PDI can eradicate bacteria, its effectiveness could be limited by hidden intraosseous biofilms around dental implants that are sheltered from direct illumination. To address these secluded biofilm formations, we envision the infected implant to function as an optical waveguide that precisely delivers the needed light intensity to the intraosseous biofilm and destructs the bacteria in PDI. To test this potential application, we conducted fundamental optical experiments on the light transmitting properties of ZrO_2_ dental implants. Further, we combined PDI with ZrO_2_ discs as optical waveguides to test the effectiveness of eradicating *S. epidermidis* biofilm in a first in vitro setup. Findings were compared to PDI that was simultaneously performed with non-transparent TAV control discs. As for the current research in this field no study has yet combined these two experimental approaches. Thus, allowing for a comprehensive data acquisition of ZrO_2_ dental implants as optical waveguides in PDI.

It was shown that light transmission through ZrO_2_ dental implants depends on the illumination wavelength with a maximum transmission at 882 nm [37]. The MB photosensitizer used for the PDI has an excitation maximum at 664 nm which is close to 882 nm [29]. While light transmission is nearly maximized at 664 nm, using a different photosensitizer with a maximum excitation closer to the peak transmission of 882 nm may further increase the waveguiding capacity of ZrO_2_ implants. A tetra pyrrole structured photosensitizer (i.e., zinc Pc derivative) with an excitation maximum at 690 nm is an option here to test in future experiments [24]. As the maximum excitation of the MB used in the in vitro PDI experiments is 664 nm, the average light intensity transmission through ZrO_2_ dental implants in the red-light range (650–675 nm) was more closely assessed. The results showed an increasing proportion of light intensity being transmitted with increasing implant diameter.

The additionally observed disproportionate increase in light transmission relative to the implants cross-sectional area could potentially be ascribed to the occurrence of light reflection and scattering. Such physical phenomena might influence the measured light intensity behind the implant. In an in vivo setting such light processes are thought to be even more predominant as differences in tissue composition around the illuminated area increase reflection and scattering [38]. Thus, the waveguiding function of ZrO_2_ dental implants is thought to be significantly improved in an in vivo setup as physical light processes from different tissue compositions come to play. Future research will need to determine the exact effect of tissue composition on waveguiding function and related PDI outcome in PI treatment.

In the next step, the light polarization through ZrO_2_ dental implants was investigated. No polarization was found, and the transmitted red-light intensity remained constant. This indicates an advantage over polarizing materials because the influence of electromagnetic oscillation direction can be neglectable. As a result, the illumination setup in a potential clinical PDI application could be adapted more dynamically without optical restrictions stemming from polarization. Another significant advantage for targeting hidden intraosseous biofilms might be the observed homogenous light dispersion [17]. While injection of MB into the surrounding tissue could reach the hidden biofilms, sufficient direct red-light illumination may be impossible in some patients due to anatomical features [39]. A ZrO_2_ implant with homogenous light transmission to all adjacent tissues would guarantee inactivation of the complete biofilm around the dental implant. It is also envisioned that the development of new bioceramic materials, as well as remodeling of the dental implant’s microstructure, could influence the light-scattering feature, therefore improving the guiding of the transmitted light intensity to the hard-to-reach intraosseous biofilm formations [40,41]. The ZrO_2_ light-transmission feature, combined with the mentioned reflection and scattering in adjacent tissue, displays advantageous characteristics that could increase the PDI effectiveness in a potential in vivo setup.

The conducted in vitro PDI experiment focused on eradicating *S. epidermidis* biofilms instead of planktonic bacteria cultures, as the former are known to be a key component of the PI pathogenesis [16]. While planktonic bacteria cultures are known to be susceptible to established antibiotics, biofilms demonstrate higher antibiotic tolerance [42]. Biofilms are also known to induce AMR formation, potentially leading to treatment failure under common antibiotics [42,43,44]. In contrast, PDI is not known to cause any AMR formations, leading to an alternative treatment option to target this clinical problem [45]. Therefore, this pilot study investigated the effectiveness of PDI in destructing bacterial biofilm.

The conducted in vitro PDI experiments on established *S. epidermidis* biofilms around ZrO_2_ and TAV discs demonstrated a strong reduction in CFU_rel_ and a significant advantage of ZrO_2_ over TAV. Light-transmitting properties of ZrO_2_ enable electromagnetic waves to penetrate ZrO_2_ discs, leading to the envisioned destruction of biofilm on the opposite disc part [46]. Thus, PDI on ZrO_2_ discs shows greater CFU reduction than on non-transparent TAV discs. This observation holds true for ZrO_2_ over all light intensities tested, despite no significant differences from lowest to higher intensities. The advantage of ZrO_2_ over TAV was especially pronounced with 85% CFU reduction at 1 μg/mL MB and 15 J/cm^2^ light intensity.

While bacteria biofilms show varying response rates to established antibiotic treatment protocols with up to 70% survival, the obtained CFU reduction under PDI suggests a strong bactericidal effect in comparison [43]. It is noted that even the smallest MB concentration of 1 µg/mL demonstrated a significant CFU reduction on ZrO_2_ discs. The waveguiding function of ZrO_2_ discs might enable even small MB concentrations to be effectively activated for ROS formation, as the needed light energy is sufficiently transmitted. This minute MB concentration stands in contrast to preexisting studies in the field of antibacterial PDI that involve MB concentrations reaching up to 200 µg/mL [47,48,49]. High MB concentrations increase the rate of bactericidal ROS formation, destroying prokaryotic cells. However, ROS also show cytotoxic effects on human cell lines, and high MB concentrations might therefore risk undesired human tissue lesions [50]. Thus, a low MB concentration, as shown in this pilot study, is an effective option to reduce CFU. It would minimize biochemical side-effects on vital human tissues induced by overdosed photosensitizer concentrations [51].

The waveguiding function of ZrO_2_ enabled this study to combine the low MB concentration with a relatively small light intensity of visible red light. The in vitro PDI experiments showed a significant CFU reduction with light intensities as low as 15 and 30 J/cm^2^. Existing antibacterial PDI studies on *Staphylococci* species needed to implement 4-fold higher light intensities to obtain similar CFU reduction rates [52]. Those higher light intensities risk adverse side effects, such as local overheating and subsequent tissue damage [53]. The implementation of non-ionizing electromagnetic waves in the conducted PDI is another important aspect to mention in this pilot study. The implemented light source does not cause carcinogenic DNA damage [54]. Therefore, our study demonstrated that effective PDI is possible without the risk of thermal overheating or induction of DNA mutations and, as a result, could reduce the stress on surrounding tissue.

Benefits of those findings could be seen in PI pathogenesis, as well as in the prevention thereof. With increasing implementation of bio-ceramic implants in other medical fields as well, the potential application spectrum for PDI supported by light-transmitting materials may even be expanded to different anatomical regions [55]. Thus, PDI, as an alternative to antibiotics, could help combat the spread of AMR. In line with the WHO’s One Health approach, PDI exhibits a broad perspective, with potential applications in other medical fields.

Despite these results, this study has a few limitations influencing possible conclusions. We aimed to effectively cultivate bacterial biofilms on ZrO_2_ and TAV discs for subsequent PDI treatment. Focusing on a single *S. epidermidis* strain with its known biofilm formation activity represented a feasible model for the conducted experiments. However, intraoral biofilms on dental implants involve multiple bacteria species, and continuing research is needed to imitate those complex biofilm conditions in future in vitro PDI experiments. Further, the PDI experiments were limited to three different MB concentrations and the two presented light intensities. This results in a restricted number of treatment combinations, only spanning a selected range of possibilities. The aim of this pilot study was to investigate the fundamental feasibility of such a PDI approach and gain the first understanding of a potential clinical application. Future studies may use those treatment combinations as guidance for more in-depth research into the optimal MB and light-intensity combination. While this study focused on one single PDI session, serial applications of PDI might present another option to enhance the reduction of bacterial biofilms in future experiments. Additionally, the study setup did not allow for the vital bacteria concentration on the discs to be determined before the PDI. This limitation was specifically targeted by working with H_2_O treatment control groups that simulated the bacterial growth rate without MB and PDI exposure. This growth rate was used as a reference to determine the CFU_rel_ after the PDI procedure. Another aspect is the PDI-induced cell toxicity of human cells. Due to the implemented low MB concentration in the first in vitro setup, this study did not focus on determining the extent to which eukaryotic cells take damage through produced ROS. However, future research in the field of PDI clinical application should investigate this aspect to guarantee a safe treatment for patients suffering from PI.

## 5. Conclusions

This pilot study conducted fundamental research for a potential PDI treatment of PI, relying on optically waveguided ZrO_2_ dental implants. Optical experiments on ZrO_2_ dental implants were combined with in vitro PDI destruction of *S. epidermidis* biofilms grown around ZrO_2_ and TAV discs to determine the possible bactericidal effect. ZrO_2_ dental implants demonstrated notable waveguiding properties. Further, ZrO_2_ discs showed significant advantages over TAV discs in the in vitro PDI biofilm destruction. PDI effectiveness depended on photosensitizer concentration (I) and illumination light intensity (II), with an achieved maximum bactericidal effect of 85% CFU reduction. With respect to the pressing need for global AMR reduction, PDI presents a promising augmentative treatment option for PI in line with the WHO One Health approach.

## Data Availability

The data presented in this study are available in the Supplementary Materials.

## Supplementary Materials

The following supporting information can be downloaded at https://www.mdpi.com/article/10.3390/microorganisms13040850/s1, Figure S1: Preliminary investigations on ZrO_2_ and TAV discs depicting perpendicular illumination from above with a red-light laser diode pointer (wavelength: 640-660 nm and output < 1 mW). Figure S2: Optical investigation setup from Figure S1 with perpendicular illumination from the side. Figure S3: Preliminary investigations on ZrO_2_ dental implants. Figure S4: Transmitted red-light intensity divided by the cross-sectional area of the corresponding ZrO_2_ diameter. Figure S5: PDI with 10 µg/mL MB of *Staphylococcus epidermidis* biofilm formations on ZrO_2_ and TAV discs with additional light intensities 45 and 90 J/cm^2^. Table S1: Underlying data for Figure 4B. Table S2: Underlying data for Figure S5. Table S3: Underlying data for Figure 6. Table S4: Underlying raw data for Figure 6.

## Abbreviations

The following abbreviations are used in this manuscript:

AMR	antimicrobial resistance
MB	Methylene Blue
PDI	Photodynamic Inactivation
PI	periimplantitis
ROS	reactive oxygen species
TAV	titanium alloy Ti-6AI-4V
ZrO2	zirconium dioxide ceramics
