# Supplementary material for: Zirconium Dental Implants as Potential Optical Waveguides in Photodynamic Inactivation of Bacterial Biofilms—A Pilot Study
Source: Microorganisms. 2025 Apr 8;13(4):850. doi: 10.3390/microorganisms13040850 (PMC12029833; doi:10.3390/microorganisms13040850)
Supplement: Supplementary file 1 [file microorganisms-13-00850-s001.zip › microorganisms-3526020-supplementary.pdf]

Supplementary Material for "Zirconium Dental Implants as Potential Optical Waveguides in Photodynamic Inactivation of Bacterial Biofilms – A Pilot Study"

Supplementary Figures:

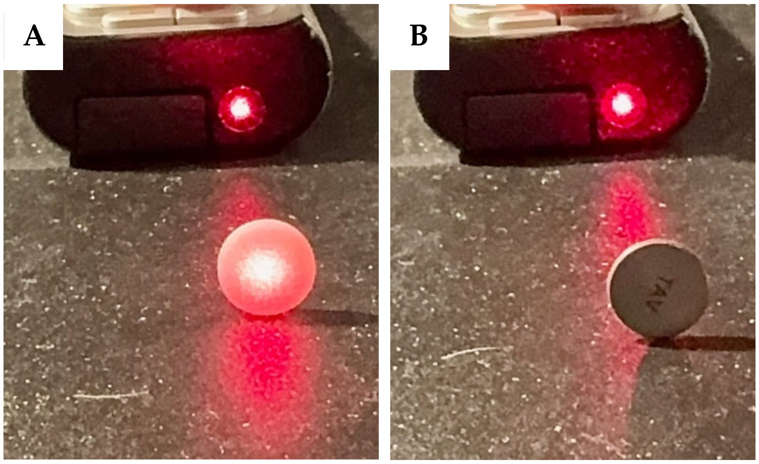

Figure S1. Preliminary investigations on ZrO_2 and TAV discs depicting perpendicular illumination from above with a red-light laser diode pointer (wavelength: 640-660 nm and output < 1 mW). A ZrO_2 disc with a diameter of 10 mm showing strong red-light penetration (**A**). In comparison, a TAV disc with the same proportions allowed no red-light penetration (**B**). ZrO_2 – Zirconium dioxide ceramics; TAV- Titanium alloy Ti-6AI-4V.

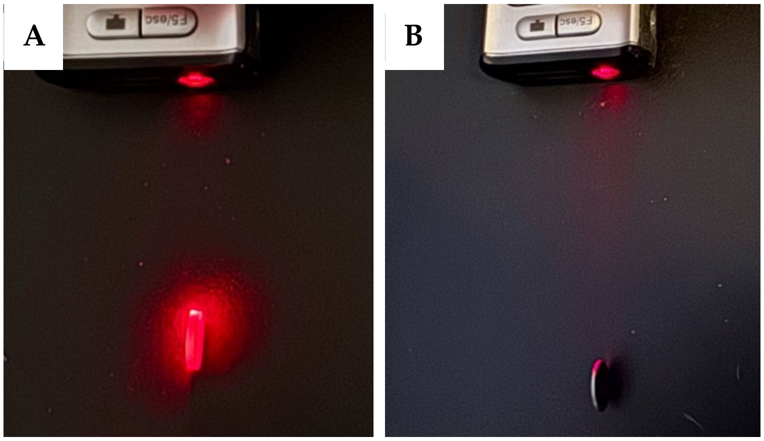

Figure S2. Optical investigation setup from **Figure S1** with perpendicular illumination from the side. The ZrO_2 disc showing red-light penetration and scattering (**A**). The TAV disc depicting neither penetration nor scattering (**B**). ZrO_2 – Zirconium dioxide ceramics; TAV- Titanium alloy Ti-6AI-4V.

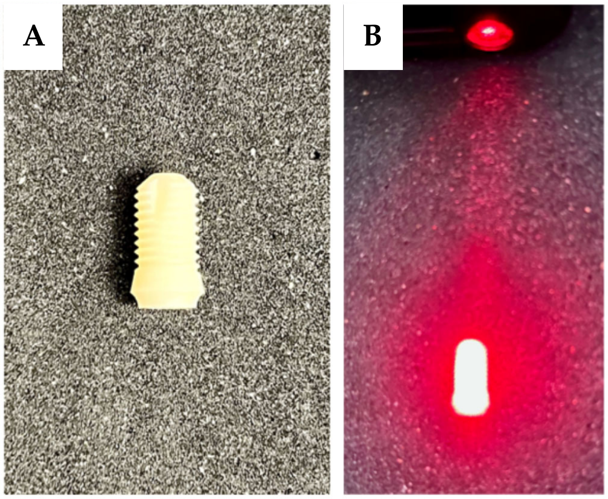

Figure S3. Preliminary investigations on ZrO_2 dental implants. A ZrO_2 implant in daylight (length: 8 mm) (**A**). The same implant illuminated with a red-light laser diode pointer (wavelength: 640-660 nm and output < 1 mW) depicts strong light penetration and visible scattering (**B**). First analyses of ZrO_2 discs with a PerkinElmer LAMBDA 1050+ UV/Vis/NIR Spectrophotometer revealed that the wavelength spectrum of penetration is 550 - 1200 nm. This possibility allows for the combination with most photosensitizers used today. ZrO_2 – Zirconium dioxide ceramics.

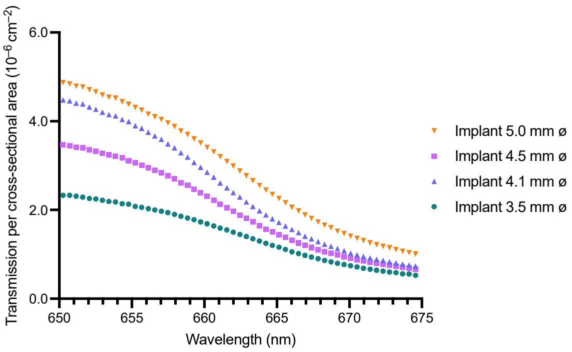

(**A**)

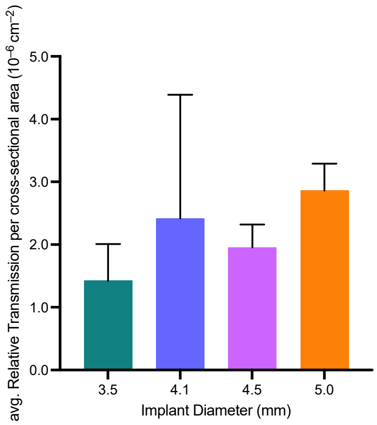

(**B**)

Figure S4. Transmitted red-light intensity divided by the cross-sectional area of the corresponding ZrO_2 diameter (**A**). Although normalized to its cross-sectional area, the four graphs largely differ at 650 nm and converge towards 675 nm. The corresponding average transmitted red-light intensity between 650-675 nm for each implant (**B**). The light transmission correlates with the diameter and shows a positive relationship between average light transmission value and cross-sectional area. ZrO_2 – Zirconium dioxide ceramics.

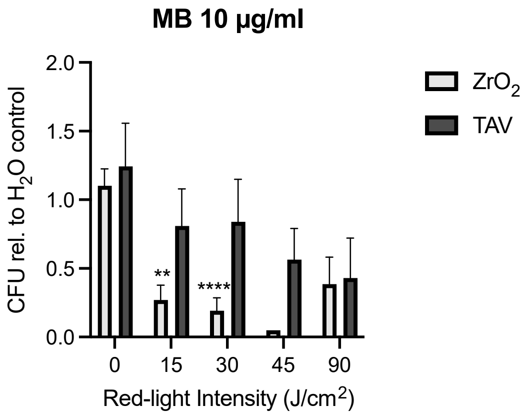

Figure S5. PDI with 10 µg/ml MB of *Staphylococcus epidermidis* biofilm formations on ZrO_2 and TAV discs with additional light intensities 45 and 90 J/cm². A strong CFU_{rel} reduction is seen in the ZrO_2 group indicating a trend of increasing light intensity leading to increased bactericidal effect. Statistical analysis was performed with the Welch Two Sample t-test with asterisks indicating statistically significant p-values for CFU reduction (**; $p < 0.01$, ****; $p < 0.0001$). PDI – Photodynamic Inactivation; MB – Methylene Blue; ZrO_2 – Zirconium dioxide ceramics; TAV – Titanium alloy Ti-6AI-4V.

Supplementary Tables:

Implant ø (mm)	Avg. Relative intensity transmission 650-675 nm (10^{-6}) Repl. 1	Repl. 2
3.5	0.322	0.772
4.1	2.32	0.239
4.5	1.48	1.01
5	1.91	2.59

Table S1. Underlying data for **Figure 4B**.

MB 10 µg/ml	ZrO_2				TAV			
Red-light intensity (J/cm²)	Mean CFU_{rel}	Std.	n	p-value	Mean CFU_{rel}	Std.	n	p-value
0	1.10	0.37	9		1.24	1.04	11	
15	0.27	0.30	8	0.000132	0.81	0.89	11	0.31
30	0.19	0.23	6	0.000054	0.84	0.88	8	0.37
45	0.05	0.00	1	nd.	0.56	0.39	3	nd.
90	0.39	0.28	2	nd.	0.43	0.50	3	nd.

Table S2. Underlying data for **Figure S5.** Depicting the obtained CFU_{rel}, standard deviation, number of technical replicates and calculated p-value indicated with asterisks in **Figure S5.** ZrO_2 – Zirconium dioxide ceramics; TAV – Titanium alloy Ti-6AI-4V; Std. – standard deviation; n – number of technical replicates; nd. – not defined.

MB 1 µg/ml	ZrO$_2$				TAV			
Red-light intensity (J/cm^2)	Mean CFU$_{rel}$	Std.	n	p-value	Mean CFU$_{rel}$	Std.	n	p-value
0	1.30	0.78	6		0.99	0.99	6	
15	0.15	0.13	6	0.01483	0.54	0.32	6	0.33
30	0.37	0.35	6	0.03197	0.73	0.74	6	0.62

MB 10 µg/ml	ZrO$_2$				TAV			
Red-light intensity (J/cm^2)	Mean CFU$_{rel}$	Std.	n	p-value	Mean CFU$_{rel}$	Std.	n	p-value
0	1.10	0.37	9		1.24	1.04	11	
15	0.27	0.30	8	0.000132	0.81	0.89	11	0.31
30	0.19	0.23	6	0.000054	0.84	0.88	8	0.37

MB 100 µg/ml	ZrO$_2$				TAV			
Red-light intensity (J/cm^2)	Mean CFU$_{rel}$	Std.	n	p-value	Mean CFU$_{rel}$	Std.	n	p-value
0	0.86	0.64	11		1.09	1.12	6	
15	0.27	0.35	11	0.01741	0.22	0.29	6	0.12
30	0.24	0.38	11	0.01298	0.28	0.39	6	0.15

Table S3. Underlying data for **Figure 6**. Depicting the obtained mean CFU$_{rel}$, standard deviation, number of technical replicates and calculated p-value indicated with asterisks in **Figure 6**. ZrO$_2$ – Zirconium dioxide ceramics; TAV – Titanium alloy Ti-6AI-4V; Std. – standard deviation; n – number of technical replicates.

Zirconium (ZrO$_2$)

MB 1 µg/ml	CFU relative to H$_2$O control (CFU$_{rel}$)					
Red-light intensity (J/cm^2)	*Repl. 1*	*Repl. 2*	*Repl. 3*	*Repl. 4*	*Repl. 5*	*Repl. 6*
0	2.68421	1.15385	0.76893	0.99092	1.66667	0.53333
15	0.28056	0.26327	0.01324	0.26418	0.07976	0.01100
30	0.79839	0.06208	0.22238	0.04066	0.81818	0.25333

MB 10 µg/ml	CFU relative to H$_2$O control (CFU$_{rel}$)								
Red-light intensity (J/cm^2)	*Repl. 1*	*Repl. 2*	*Repl. 3*	*Repl. 4*	*Repl. 5*	*Repl. 6*	*Repl. 7*	*Repl. 8*	*Repl. 9*
0	1.71711	1.05128	0.71654	0.88462	0.97727	1.00704	0.76877	1.72222	1.08333
15	0.46389	0.51429	0.03059	0.01100	0.31622	0.80083	0.00023	0.03200	
30	0.46855	0.00004	0.02989	0.15797	0.48864	0.01067			

MB 100 µg/ml	CFU relative to H$_2$O control (CFU$_{rel}$)										
Red-light intensity (J/cm^2)	*Repl. 1*	*Repl. 2*	*Repl. 3*	*Repl. 4*	*Repl. 5*	*Repl. 6*	*Repl. 7*	*Repl. 8*	*Repl. 9*	*Repl. 10*	*Repl. 11*
0	0.15698	1.63158	0.50220	2.34868	0.62821	0.89338	0.59341	0.78462	0.12960	0.97222	0.83333
15	0.02261	0.28190	0.15724	0.43333	0.38367	0.09381	0.00026	1.24367	0.27896	0.02190	0.09250
30	0.04020	0.00231	0.10645	1.32258	0.24583	0.20003	0.01416	0.00350	0.08283	0.31818	0.25000

Titanium (TAV)

MB 1 µg/ml	CFU relative to H$_2$O control (CFU$_{rel}$)					
Red-light intensity (J/cm^2)	*Repl. 1*	*Repl. 2*	*Repl. 3*	*Repl. 4*	*Repl. 5*	*Repl. 6*
0	0.38776	0.62899	0.45588	2.61146	1.78080	0.07882
15	0.23179	0.40071	0.45946	1.13939	0.64087	0.39583
30	0.21333	0.39402	0.35037	0.32293	0.95385	2.15079

MB 10 µg/ml	CFU relative to H$_2$O control (CFU$_{rel}$)									
Red-light intensity (J/cm^2)	*Repl. 1*	*Repl. 2*	*Repl. 3*	*Repl. 4*	*Repl. 5*	*Repl. 6*	*Repl. 7*	*Repl. 8*	*Repl. 9*	*Repl. 10*
0	0.20449	0.39602	3.61765	0.28005	1.65000	0.56667	0.99359	2.32967	1.22101	0.68273
15	0.00208	0.00820	0.40476	2.45455	1.26667	0.37273	2.43243	0.94242	0.50000	0.46078
30	0.11867	0.11413	0.98629	0.97059	0.67951	0.08333	2.75079	1.00833		

MB 100 µg/ml	CFU relative to H$_2$O control (CFU$_{rel}$)					
Red-light intensity (J/cm^2)	*Repl. 1*	*Repl. 2*	*Repl. 3*	*Repl. 4*	*Repl. 5*	*Repl. 6*
0	0.27532	0.71790	1.55882	3.12245	0.73551	0.10209
15	0.00031	0.05839	0.16216	0.12227	0.80357	0.14583
30	0.89000	0.05471	0.07305	0.00155	0.66154	0.02192

Table S4. Underlying raw data for **Figure 6.**
